# Constraining *f*(*T*) teleparallel gravity by big bang nucleosynthesis: *f*(*T*) cosmology and BBN

**DOI:** 10.1140/epjc/s10052-017-5143-8

**Published:** 2017-08-31

**Authors:** S. Capozziello, G. Lambiase, E. N. Saridakis

**Affiliations:** 1 Dipartimento di Fisica “E. Pancini”, Università di Napoli “Federico II”, Complesso Universitario di Monte Sant’Angelo, Edificio G, Via Cinthia, 80126 Napoli, Italy; 2 Istituto Nazionale di Fisica Nucleare (INFN) Sezione di Napoli, Complesso Universitario di Monte Sant’Angelo, Edificio G, Via Cinthia, 80126 Napoli, Italy; 3 Gran Sasso Science Institute, Viale F. Crispi, 7, 67100 L’Aquila, Italy; 4 Dipartimento di Fisica E.R. Cainaiello, University of Salerno, Via Giovanni Paolo II, 84084 Fisciano, SA Italy; 5 INFN, Gruppo Collegato di Salerno, Sezione di Napoli, Via Giovanni Paolo II, 84084 Fisciano, SA Italy; 6 Department of Physics, National Technical University of Athens, Zografou Campus, 157 73 Athens, Greece; 7 CASPER, Physics Department, Baylor University, Waco, TX 76798-7310 USA

## Abstract

We use Big Bang Nucleosynthesis (BBN) observational data on the primordial abundance of light elements to constrain *f*(*T*) gravity. The three most studied viable *f*(*T*) models, namely the power law, the exponential and the square-root exponential are considered, and the BBN bounds are adopted in order to extract constraints on their free parameters. For the power-law model, we find that the constraints are in agreement with those obtained using late-time cosmological data. For the exponential and the square-root exponential models, we show that for reliable regions of parameters space they always satisfy the BBN bounds. We conclude that viable *f*(*T*) models can successfully satisfy the BBN constraints.

## Introduction

Cosmological observations coming from Type Ia Supernovae [1, 2], cosmic microwave background radiation [3, 4] and the large scale structure [5, 6] provide evidence that the Universe is currently in an accelerating phase. This result is, in general, ascribed to the existence of a sort of dark-energy (DE) sector in the Universe, an exotic energy source characterized by a negative pressure. At late times, the dark-energy sector eventually dominates over the cold dark matter (CDM), and it drives the Universe to the observed accelerating expansion.

A possibility that can be explored to explain the accelerated phase of the Universe is to consider a theory of gravity based on the Weitzenböck connection, instead of the Levi-Civita one, which deduces that the gravitational field is described by the torsion instead of the curvature tensor. In such theories, the torsion tensor is achieved from products of first derivatives of tetrad fields, and hence no second derivatives appear. This *teleparallel* approach [7, 8], is closely related to general relativity, except for “boundary terms” [9, 10] that involve total derivatives in the action, and thus one can construct the Teleparallel Equivalent of General Relativity (TEGR), which is completely equivalent with general relativity at the level of equations but is based on torsion instead of curvature. Hence, one can start from TEGR and construct various gravitational modifications based on torsion, with *f*(*T*) gravity being the most studied one [11–13]. In particular, it may represent an alternative to inflationary models without the use of the inflaton, as well as to effective DE models, in which the Universe acceleration is driven by the extra torsion terms [11–39] (for a detailed review, see [40]). The main advantage of *f*(*T*) gravity is that the field equations are of second order, a property that makes these theories simpler if compared to the dynamical equations of other extended theories of gravity, such as *f*(*R*) gravity in metric formalism. Moreover, we point out the possibility to recover specific form of *f*(*T*) model by making use of a tomographic approach capable of addressing the form of the *f*(*T*) function at any epoch. As discussed, in [41, 42], a tomographic description for cosmological models is, in principle, always possible ranging from the primordial quantum states up to the today accelerated epoch. The consistency of the approach strictly relies on the available observational data sets that should be matched with theoretical models at various epochs, i.e. at various redshift [43]. A fundamental role in this perspective is played by the BBN, which constitutes a formidable and independent constraint for any cosmological model.

The aim of this paper is to explore the implications of *f*(*T*) gravity to the formation of light elements in the early Universe, i.e. to the BBN, exploring the possibility to constrain *f*(*T*) models (here considered) by BBN observational data. BBN has occurred between the first fractions of second after the big bang, around ${\sim } 0.01$ s, and a few hundreds of seconds after it, when the Universe was hot and dense (indeed BBN, together with cosmic microwave background radiation, provides the strong evidence about the high temperatures characterizing the primordial Universe). It describes the sequence of nuclear reactions that yielded the synthesis of light elements [44, 45], and therefore drove the observed Universe. In general, from BBN physics, one may infer stringent constraints on a given cosmological model. Hence, in this work, we shall confront various *f*(*T*) gravity models with BBN calculations based on current observational data on the primordial abundance of ${}^4\mathrm{He}$, and we shall extract constraints on their free parameters. We shall refer to specific *f*(*T*) models that mimic $\Lambda$CDM models, but generally models of gravity in which torsion field is present are investigated assuming the torsion coupled to matter field [46].

The layout of the paper is as follows. In Sect. 2 we review *f*(*T*) gravity and the related cosmological models. In Sect. 3 we use BBN calculations in order to impose constraints on the free parameters of specific *f*(*T*) gravity models. Conclusions are reported in Sect. 4.

## *f*(*T*) gravity and cosmology

In teleparallel gravity, one adopts the curvatureless Weitzenböck connection (contrarily to general relativity, which is based on the torsion-less Levi-Civita connection), which gives rise to the non-null torsion tensor:

1 $$\begin{aligned} T^\lambda _{\mu \nu }=\hat{\Gamma }^\lambda _{\nu \mu }-\hat{\Gamma }^\lambda _{\mu \nu } =e^\lambda _i(\partial _\mu e^i_\nu - \partial _\nu e^i_\mu ), \end{aligned}$$

where $e^i_\mu (x)$ are the vierbein fields defined as $g_{\mu \nu }(x)=\eta _{ij} e^i_\mu (x)e^j_\nu (x)$. Remarkably, the torsion tensor (1) encompasses all the information as regards the gravitational field. The Lagrangian density is built using its contractions, and hence the teleparallel action is given by

2 $$\begin{aligned} I = \frac{1}{16\pi G}\int \mathrm{d}^4x e T, \quad T={S_\rho }^{\mu \nu }{T^\rho }_{\mu \nu }, \end{aligned}$$

where $e=det(e^i_\mu )=\sqrt{-g}$, *T* is the torsion scalar

3 $$\begin{aligned} {S_\rho }^{\mu \nu }= & {} \frac{1}{2}({K^{\mu \nu }}_\rho +\delta ^\mu _\rho {T^{\theta \nu }}_\theta -\delta ^\nu _\rho {T^{\theta \mu }}_\theta ) \end{aligned}$$

4 $$\begin{aligned} {K^{\mu \nu }}_\rho= & {} -\frac{1}{2}({T^{\mu \nu }}_\rho -{T^{\nu \mu }}_\rho -{T_\rho }^{\mu \nu }), \end{aligned}$$

with ${K^{\mu \nu }}_\rho$ the contorsion tensor, which gives the difference between Weitzenböck and Levi-Civita connections. One can now start from TEGR, and generalize action (2) in order to construct gravitational modifications based on torsion. The simplest scenario is to consider a Lagrangian density that is a function of *T*, namely

5 $$\begin{aligned} I = \frac{1}{16\pi G}\int {\mathrm{d}^4xe[T+f(T)]}, \end{aligned}$$

which reduces to TEGR as soon as $f(T)=0$. In order to explore the cosmological implications of *f*(*T*) gravity, we focus on homogeneous and isotropic geometry, considering the usual choice for the vierbeins $e_{\mu }^A=\mathrm{diag}(1,a,a,a)$, which corresponds to a flat Friedmann–Robertson–Walker (FRW) background metric of the form $\mathrm{d}s^2= \mathrm{d}t^2-a^2(t)\,\delta _{ij} \mathrm{d}x^i \mathrm{d}x^j$, where *a*(*t*) is the scale factor. Equations (1), (3) and (4) allow one to derive a relation between the torsion *T* and the Hubble parameter ${H=\frac{\dot{a}}{a}}$, namely

6 $$\begin{aligned} T=-6H^2. \end{aligned}$$

Hence, in the case of FRW geometry, and assuming that the matter sector corresponds to a perfect fluid with energy density $\rho$ and pressure *p*, the cosmological fields equations read [40]

7 $$\begin{aligned} 12H^2[1+f']+[T+f]=16\pi G\rho , \end{aligned}$$

$$\begin{aligned} 48H^2f''\dot{H}-(1+f')[12H^2+4\dot{H}]-(T-f)=16\pi G p, \end{aligned}$$

where $f'=\mathrm{d}f/\mathrm{d}T$. The equations close by considering the equation of continuity for the matter sector, namely $\dot{\rho }+3H(\rho +p)=0$. One can rewrite Eq. (7) in the usual form

8 $$\begin{aligned} H^2= & {} \frac{8\pi G}{3}(\rho +\rho _T), \end{aligned}$$

9 $$\begin{aligned} 2\dot{H}+3H^2= & {} -\frac{8\pi G}{3}(p+p_T), \end{aligned}$$

where

10 $$\begin{aligned} \rho _T= & {} \frac{3}{8\pi G}\left[ \frac{Tf'}{3}-\frac{f}{6}\right] ,\end{aligned}$$

11 $$\begin{aligned} p_T= & {} \frac{1}{16\pi G}\, \frac{f-T f'+ 2T^2f''}{1+f'+ 2{T}f''} \end{aligned}$$

are the effective energy density and pressure arising from torsional contributions. One can therefore define the effective torsional equation-of-state parameter as $\omega _{T}\equiv \frac{p_T}{\rho _T}$, so that in these classes of theories, the effective torsional terms are responsible for the accelerated phases of the early and/or late Universe [40].

Let us present now three specific *f*(*T*) forms, which are the viable ones amongst the variety of *f*(*T*) models with two parameters out of which one is independent, i.e., which pass the basic observational tests [48].

1. The power-law model by Bengochea and Ferraro (hereafter $f_{1}$CDM) [12] is characterized by the form
  12 $$\begin{aligned} f(T) = \beta |T|^n, \end{aligned}$$
  where $\beta$ and *n* are the two model parameters. Inserting this *f*(*T*) form into Friedmann equation (7) at present, we obtain
  13 $$\begin{aligned} \beta =(6H_0^2)^{1-n}\frac{\Omega _{m0}}{2n-1}, \end{aligned}$$
  where ${\Omega _{m0}=\frac{8\pi G \rho _{m}}{3H_0^2}}$ is the matter density parameter at present, and
  $$\begin{aligned} H_0= & {} 73.02\pm 1.79\,{\text {km/(sMpc)}} \\\sim & {} 2.1 \times 10^{-42}\,\text{ GeV } \end{aligned}$$
  is the current Hubble parameter value. The best fit on the parameter *n* is obtained taking the $\mathrm{CC}+\mathrm{H}_0+\mathrm{SNeIa}+\mathrm{BAO}$ observational data, and it reads [49]
  14 $$\begin{aligned} n=0.05536. \end{aligned}$$
  Clearly, for $n=0$ the present scenario reduces to $\Lambda$CDM cosmology, namely ${T}+f(T)=T-2\Lambda$, with $\Lambda =-\beta /2$.
2. The Linder model (hereafter $f_{2}$CDM) [13] arises from
  15 $$\begin{aligned} f(T)=\alpha T_{0}\big (1-e^{-p\sqrt{T/T_{0}}}\big ),\quad p=\frac{1}{b}, \end{aligned}$$
  with $\alpha$ and *p* (*b*) the two model parameters. In this case (7) gives
  16 $$\begin{aligned} \alpha =\frac{\Omega _{m0}}{1-(1+p)e^{-p}}. \end{aligned}$$
  The $\mathrm{CC}+\mathrm{H}_0+\mathrm{SNeIa}+\mathrm{BAO}$ observational data imply that the best fit of *b* is [49]
  17 $$\begin{aligned} b=0.04095. \end{aligned}$$
  As we can see, for $p \rightarrow +\infty$ the present scenario reduces to $\Lambda$CDM cosmology.
3. Motivated by exponential *f*(*R*) gravity [50], Bamba et al. introduced the following *f*(*T*) model (hereafter $f_{3}$CDM) [18]:
  18 $$\begin{aligned} f(T)=\alpha T_{0}(1-e^{-pT/T_{0}}), \quad p=\frac{1}{b}, \end{aligned}$$
  with $\alpha$ and *p* (*b*) the two model parameters. In this case we obtain
  19 $$\begin{aligned} \alpha =\frac{\Omega _{m0}}{1-(1+2p)e^{-p}}. \end{aligned}$$
  For this model, and using $\mathrm{CC}+\mathrm{H}_0+\mathrm{SNeIa}+\mathrm{BAO}$ observational data, the best fit is found to be [49]
  20 $$\begin{aligned} b=0.03207. \end{aligned}$$
  Similarly to the previous case we can immediately see that $f_{3}$CDM model tends to $\Lambda$CDM cosmology for $p \rightarrow +\infty$.

The above *f*(*T*) models are considered viable in the literature because they pass the basic observational tests [40]. They are characterized by two free parameters. Notice that one could also construct *f*(*T*) models with more than two parameters, for example, combining the above scenarios. However, considering many free parameters would be a significant disadvantage concerning the corresponding values of the information criteria.

## Big bang nucleosynthesis in *f*(*T*) cosmology

In the section, we examine the BBN in the framework of *f*(*T*) cosmology. As is well known, BBN occurs during the radiation dominated era. The energy density of relativistic particles filling up the Universe is given by ${ \rho =\frac{\pi ^2}{30}g_* \mathcal{T}^4}$, where $g_*\sim 10$ is the effective number of degrees of freedom and $\mathcal{T}$ the temperature. The neutron abundance is computed via the conversion rate of protons into neutrons. The total rate reads

21 $$\begin{aligned} \Lambda (\mathcal{T}) =4 A\, \mathcal{T}^3(4! \mathcal{T}^2+2\times 3! \mathcal{Q}\mathcal{T}+2! \mathcal{Q}^2), \end{aligned}$$

where $\mathcal{Q}=m_n-m_p$ is the mass difference of neutron and proton, and $A=1.02 \times 10^{-11}$ GeV$^{-4}$. The primordial mass fraction of ${}^4 \mathrm{He}$ can be estimated by making use of the relation [44]

22 $$\begin{aligned} Y_p\equiv \lambda \, \frac{2 x(t_f)}{1+x(t_f)}. \end{aligned}$$

Here $\lambda =e^{-(t_n-t_f)/\tau }$, with $t_f$ the time of the freeze-out of the weak interactions, $t_n$ the time of the freeze-out of the nucleosynthesis, $\tau =8803\pm 1.1$ s the neutron mean lifetime [47], and $x(t_f)=e^{-\mathcal{Q}/\mathcal{T}(t_f)}$ is the neutron-to-proton equilibrium ratio. The function $\lambda (t_f)$ is interpreted as the fraction of neutrons that decay into protons during the interval $t\in [t_f, t_n]$. Deviations from the fractional mass $Y_p$ due to the variation of the freezing temperature $\mathcal{T}_f$ are given by

23 $$\begin{aligned} \delta Y_p=Y_p\left[ \left( 1-\frac{Y_p}{2\lambda }\right) \ln \left( \frac{2\lambda }{Y_p} -1\right) -\frac{2t_f}{\tau }\right] \frac{\delta \mathcal{T}_f}{\mathcal{T}_f}, \end{aligned}$$

where we have set $\delta \mathcal{T}(t_n)=0$ since $\mathcal{T}_n$ is fixed by the deuterium binding energy [51–54]. A recent determination of mass fraction of ${}^4\mathrm{He}$ has been obtained by using infrared and visible ${}^4\mathrm{He}$ emission lines in 45 extragalactic HII regions. The analysis yields [55] (see also [56–59])

24 $$\begin{aligned} Y_p=0.2449\pm 0.0040. \end{aligned}$$

For our estimations we shall use (24) and therefore we shall take $|\delta Y_p| < 10^{-4}$. Inserting these into (23) one infers the upper bound

25 $$\begin{aligned} \left| \frac{\delta \mathcal{T}_f}{\mathcal{T}_f}\right| < 4.7 \times 10^{-4}. \end{aligned}$$

The scale factor evolves as $a\sim t^{1/2}$, where *t* is cosmic time. The torsional energy density $\rho _T$ is treated as a perturbation to the radiation energy density $\rho$. The relation between the cosmic time and the temperature is given by ${\frac{1}{t}\simeq \big (\frac{32\pi ^3 g_*}{90}\big )^{1/2}\frac{\mathcal{T}^2}{M_{P}} }$ (or $\mathcal{T}(t)\simeq (t/\text{ s })^{1/2}$  MeV). Furthermore, we use the entropy conservation $S\sim a^3 \mathcal{T}^3=\mathrm{constant}$. The expansion rate of the Universe is derived from (8), and can be rewritten in the form

26 $$\begin{aligned} H= & {} H_\mathrm{GR}^{(R)}\sqrt{1+\frac{\rho _T}{\rho }}=H_\mathrm{GR}+\delta H, \end{aligned}$$

27 $$\begin{aligned} \delta H= & {} \left( \sqrt{1+\frac{\rho _T}{\rho }}-1\right) H_\mathrm{GR}, \end{aligned}$$

where $H_\mathrm{GR}={\sqrt{\frac{8\pi G}{2}\rho }}$ ($H_\mathrm{GR}$ is the expansion rate of the Universe in general relativity). Thus, from the relation $\Lambda = H$, one derives the freeze-out temperature $\mathcal{T}=\mathcal{T}_f\big (1+\frac{\delta \mathcal{T}_f}{\mathcal{T}_f}\big )$, with $\mathcal{T}_f\sim 0.6$ MeV (which follows from $H_\mathrm{GR}\simeq q \mathcal{T}^5$) and

28 $$\begin{aligned} \left( \sqrt{1+\frac{\rho _T}{\rho }}-1\right) H_\mathrm{GR} = 5q \mathcal{T}_f^4 \delta \mathcal{T}_f, \end{aligned}$$

from which, in the regime $\rho _T\ll \rho$, one obtains

29 $$\begin{aligned} \frac{\delta \mathcal{T}_f}{\mathcal{T}_f}\simeq \frac{\rho _T}{\rho }\frac{H_\mathrm{GR}}{10 q \mathcal{T}_f^5}, \end{aligned}$$

with $q=4! A\simeq 9.6\times 10^{-36}$ GeV$^{-4}$. In what follows we shall investigate the bounds that arise from the BBN constraints, on the free parameters of the three *f*(*T*) models presented in the previous section. These constraint will be determined using Eqs. (29) and (10). Moreover, we shall use the numerical values $\Omega _{m0}=0.25$ and $\mathcal{T}_0=2.6\times 10^{-13}\,\text{ GeV }$, where $\mathcal{T}_0$ is the present value of CMB temperature.

**Fig. 1:**
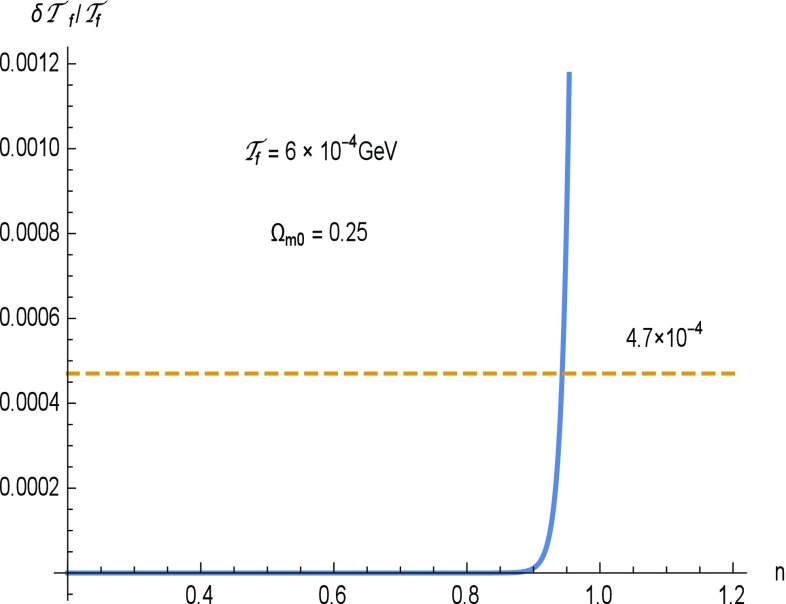
$\delta \mathcal{T}_f/\mathcal{T}_f$ from (31) vs. *n* (*thick line*) for the $f_1$CDM model of (12), and the upper bound for $\delta \mathcal{T}_f/\mathcal{T}_f$ from (25) (*dashed line*). As we can see, constraints from BBN require $n\lesssim 0.94$

1. $f_1$CDM model. For the $f_1$CDM model of (12) Eq. (10) gives
  30 $$\begin{aligned} \rho _T= & {} \frac{1}{16\pi G}[\beta (2n-1)(|6H^2|)^n] \nonumber \\= & {} \frac{3H_0^2}{8\pi G}\, \Omega _{m0}\left( \frac{\mathcal{T}}{\mathcal{T}_0}\right) ^{4n} , \end{aligned}$$
  and then (29) yields
  31 $$\begin{aligned} \frac{\delta \mathcal{T}_f}{\mathcal{T}_f}=\frac{\pi }{15}\sqrt{\frac{\pi g_*}{5}}\,\Omega _{m0}\left( \frac{\mathcal{T}_f}{\mathcal{T}_0}\right) ^{4(n-1)}\frac{1}{qM_{Pl}\mathcal{T}_f^3}. \end{aligned}$$
  In Fig. 1 we depict $\delta \mathcal{T}_f/\mathcal{T}_f$ from (31) vs. *n*, as well as the upper bound from (25). As we can see, constraints from BBN require $n\lesssim 0.94$. Remarkably, this bound is in agreement with the best fit for *n* of (14), namely $n=0.05536$, which was obtained using $\mathrm{CC}+\mathrm{H}_0+\mathrm{SNeIa}+\mathrm{BAO}$ observational data in [49].
2. $f_{2,3}$CDM model. In the case of $f_2$CDM model of (15) and $f_3$CDM model of (18), and for the purpose of this analysis, we can unified their investigation parameterizing them as
  32 $$\begin{aligned} f(T)= \alpha T_0 [1-e^{-p(T/T_0)^m}], \end{aligned}$$
  with
  $$\begin{aligned} \alpha = \frac{\Omega _{m0}}{1-(1+2mp)e^{-p}}, \end{aligned}$$
  where $$ m=\frac{1}{2}$$ for model $$f_2$$CDM and $$ m=1$$ for model $$f_3$$CDM. Inserting (32) into (29) we obtain
  33 $$\begin{aligned} \frac{\delta \mathcal{T}_f}{\mathcal{T}_f}= & {} \frac{2\pi \alpha }{15}\sqrt{\frac{\pi g_*}{5}} \left( \frac{\mathcal{T}_0}{\mathcal{T}_f}\right) ^4\frac{1}{qM_P \mathcal{T}_f^3}\nonumber \\&\times \, \left\{ \left[ mp\left( \frac{\mathcal{T}_0}{\mathcal{T}_f}\right) ^{4m}+\frac{1}{2}\right] e^{-p(\mathcal{T}_f/\mathcal{T}_0)^{4m}} -\frac{1}{2}\right\} .\nonumber \\ \end{aligned}$$
  Hence, using this relation we can calculate the value of $|\delta \mathcal{T}_f/\mathcal{T}_f|$ for various values of $p=1/b$ that span the order of magnitude of the best fit values (14) and (17) that were obtained using $\mathrm{CC}+\mathrm{H}_0+\mathrm{SNeIa}+\mathrm{BAO}$ observational data in [49], and we present our results in Table 1. As we can see, in all cases the value of $|\delta \mathcal{T}_f/\mathcal{T}_f|$ is well below the BBN bound (25). Hence, BBN cannot impose constraints on the parameter values of $f_2$CDM and $f_3$CDM models.

**Table 1:** $|\delta \mathcal{T}_f/\mathcal{T}_f|$ from (33) for different values of $p=1/b$, for $m=1/2$ ($f_2$CDM model) and $m=1$ ($f_3$CDM model)

*m*	$p=1/b$	$|\delta \mathcal{T}_f/\mathcal{T}_f|$
1/2	1	$5.723 \times 10^{-38}$
	10	$1.512 \times 10^{-38}$
	$10^2$	$1.511 \times 10^{-38}$
1	1	$1.4586 \times 10^{-37}$
	10	$1.5131 \times 10^{-38}$
	$10^2$	$1.5116 \times 10^{-38}$

## Conclusions

In this work we have investigated the *f*(*T*) model of gravity in the framework of BBN. In particular, we have examined the three most used and well studied viable *f*(*T*) models, namely the power law, the exponential and the square-root exponential, and we have confronted them with BBN calculations based on current observational data on the primordial abundance of ${}^4\mathrm{He}$. Hence, we were able to extract constraints on their free parameters.

Concerning the power-law *f*(*T*) model, the obtained constraint on the exponent *n*, is $n\lesssim 0.94$. Remarkably, this bound is in agreement with the constraints obtained using $\mathrm{CC}+\mathrm{H}_0+\mathrm{SNeIa}+\mathrm{BAO}$ observational data [49]. Concerning the exponential and the square-root exponential, we showed that, for realistic regions of free parameters, they always satisfy the BBN bounds. This means that, in these cases, BBN cannot impose strict constraints on the values of free parameters.

In summary, we showed that viable *f*(*T*) models, namely those that pass the basic observational tests, can also satisfy the BBN constraints. This feature acts as an additional advantage of *f*(*T*) gravity, which might be a successful candidate for describing the gravitational interaction. As discussed in [40], this kind of constraints could contribute in the debate of fixing the most realistic picture that can be based on curvature or torsion.
